# Clinical features at the time of non-hysteroscopic myomectomy before pregnancy, which affect adverse pregnancy outcomes: a retrospective cohort study

**DOI:** 10.1186/s12884-022-05240-7

**Published:** 2022-12-03

**Authors:** Young Ran Kim, Eun Duc Na, Jae Eun Jung, Ji Hyun Moon, Ji Yeon Lee

**Affiliations:** Department of Obstetrics and Gynecology, CHA Bundang Medical Center, CHA University School of Medicine, 13496 Seongnam, South Korea

**Keywords:** Uterine myoma, Myomectomy, Pregnancy, Obstetric outcome, Perinatal outcome

## Abstract

**Background:**

To investigate the association of clinical characteristics at the time of non-hysteroscopic myomectomy before pregnancy and adverse obstetric outcomes in the next pregnancy.

**Methods:**

In this retrospective cohort study, we identified 248 women who underwent abdominal or laparoscopic myomectomy for intramural (IM) and/or subserosal (SS) uterine myomas in Bundang CHA Medical Center before pregnancy and delivered at the same hospital between 2010 and 2020. The association between clinical characteristics at the time of myomectomy and subsequent obstetric outcomes was analyzed using the Chi-square test, the Student t-test or one-way ANOVA, and multivariable analysis.

**Results:**

There was one case of uterine rupture. The gestational age at delivery was 37.7 ± 2.4 weeks. There were 2 (0.8%) cases of fetal loss before 23 weeks, but there were no cases of perinatal death. The risk of transfusion during or after delivery was higher in the group in which multiple myomas were removed compared to the group in which only one was removed (aOR = 2.41, 95% CI [1.20–4.86], *p* = 0.014). The risk of neonatal composite morbidity was higher in the group in which myomas including the IM type were removed, than in the group in which only SS myomas were removed (aOR = 14.29, 95% CI [1.82–99.57], *p* = 0.012). Although not statistically significant, the group in which the sum of the diameters of the three largest myomas was greater than 15 cm showed a higher frequency of preterm birth (19.3% vs. 10.1%, *p* = 0.001) and lower birth weight (2901 ± 625 g vs. 3063 ± 576 g, *p* = 0.001) compared to the group with diameters less than 15 cm. Placenta accreta/increta (7.9% vs. 3.8%, *p* = 0.043) and lower placental weight (646 ± 170 g vs. 750 ± 232 g, *p* = 0.034) were more common in patients with an interval between myomectomy and pregnancy of less than 12 months compared to more than 12 months.

**Conclusions:**

To our knowledge, this is the first study to investigate the association between clinical features at the time of myomectomy before pregnancy and various adverse obstetric and perinatal outcomes. If the removed myomas are multiple, IM, large, or the interval between myomectomy and pregnancy is short, the risk of obstetric and neonatal complications may increase.

**Supplementary Information:**

The online version contains supplementary material available at 10.1186/s12884-022-05240-7.

## Background

Uterine myomas (fibroids) are the most common benign pelvic tumors found in women of childbearing age. They are monoclonal tumors of the smooth muscle cells of the myometrium. It is important to classify uterine fibroids according to whether they are located on the mucous membrane, myometrium, or serous surface of the uterus. This is because, depending on the location, the symptoms experienced by the patient are different, the treatment method is different, and the treatment result can be different [1, 2]. The International Federation of Gynecology and Obstetrics (FIGO) classification system divided a total of 9 types of fibroids, with types 0–2 submucosal fibroids and type 3 fibroids adjoining the endometrium but completely within the wall, type 4 fibroids. was defined as completely intramural myoma. Types 5 and 6 are cases in the serous layer, and type 7 represents fibromas pedunculated on the subserosal surface. Fibroids found in ectopic locations such as the cervix belong to type 8 [3].

Typical symptoms include excessive menstruation and pressure symptoms, as well as infertility in some patients. Treatment options in women of childbearing age include medications, uterine artery embolization, high-intensity focused ultrasound, and myomectomy. Myomectomy is recommended for patients with severe symptoms who wish to preserve their fertility [1]. However, there are some concerns that myomectomy may adversely affect subsequent pregnancy outcomes.

In the case of type 0–1 submucosal fibroids, which protrude into the uterine cavity, there is a high possibility of causing massive menstruation and infertility before surgical removal, but it is highly likely to improve symptoms and infertility after removal. On the other hand, type 2–5 uterine fibroids may have fewer symptoms, such as menorrhagia, compared to type 0–1, but myomectomy (hysteroscopic or laparoscopic operation or laparotomy) that includes myometrium itself may lead to poor pregnancy results [1–3].

So far, hysteroscopic resection of submucosal fibroids is known to have a strong association with complications in the subsequent pregnancy [2, 4]. However, there is still insufficient evidence regarding the risk of non-hysteroscopic myomectomy on pregnancy outcomes; therefore, it is difficult to effectively counsel women who are planning to become pregnant after a non-hysteroscopic myomectomy or those who have already undergone myomectomy. In particular, the association between the location, type, and number of resected uterine myomas, and the time interval from myomectomy to the next pregnancy with uterine rupture, placental attachment abnormality, or fetal growth in the next pregnancy is unknown.

Therefore, we investigated the association of clinical characteristics at the time of non-hysteroscopic myomectomy before pregnancy and adverse obstetric outcomes in the next pregnancy.

## Methods

This was a retrospective cohort study of 248 women who underwent abdominal or laparoscopic myomectomy for intramural (IM) and/or subserosal (SS) uterine myomas in Bundang CHA Medical Center before pregnancy and delivered at the same hospital from 2010 to 2020. Cases with submucosal myomas, those that underwent hysteroscopic myomectomy, those with maternal medical or surgical comorbidities, and those with fetal abnormalities were excluded. We reviewed the clinical characteristics at the time of myomectomy including number of uterine myomas removed, sum of the diameters of which the three biggest uterine myomas, the type of uterine myomas, and myomectomy method. In this study, 3 or fewer fibroids were removed in about 2/3 cases, so the sum of the three largest fibroid diameters was selected as a comparative measure of fibroid size. The interval time between the myomectomy and the pregnancy, maternal demographics, clinical presentations during pregnancy, laboratory examinations, ultrasound assessments, and obstetrical outcomes of all included patients were also evaluated.

Statistical analysis was performed using SPSS (version 23.0; SPSS Institute, Chicago, IL, USA) and R (R Foundation for Statistical Computing, Vienna, Austria). We analyzed discrete data using the Chi-square test, and comparisons of continuous variables were performed using the Student t-test or one-way ANOVA. We also performed a multivariable analysis including maternal age, body mass index (BMI), method of conception, and interval time between study and data collection as covariates when analyzing neonatal morbidity and mortality. Statistical significance was set at *p* < 0.05.

## Results

A total of 248 women with a history of pre-pregnancy myomectomy were analyzed. The average maternal age was 35 years, and the natural pregnancy rate was 75.4%. One, two, three and four or more fibroids were removed in 41.1%, 16.9%, 10.9%, and 31% of cases, respectively. Only the SS type was removed in 31% of cases. The time interval until the next pregnancy was less than 6 months in 18.5%, between 6 months and less than 12 months in 17.3%, between 12 months and less than 24 months in 30.6%, and more than 24 months in 33.5% of cases (Table 1). The mean gestational age at delivery was 37.7 ± 2.4 weeks. The proportion of preterm births before 37 weeks was 12.1%. There were 2 (0.8%) cases of fetal loss before 23 weeks. There was one case of uterine rupture, but there were no cases of perinatal death. The rate of admission to the neonatal intensive care unit was 16.5% (Table 2). The risk of transfusion during or after delivery was higher in the group in which multiple myomas were removed compared to the group in which only one myoma was removed (aOR = 2.41, 95% CI [1.20–4.86], *p* = 0.014) (Table 3). Although not statistically significant, the group in which the sum of the diameters of the three largest myomas was greater than 15 cm showed a higher frequency of preterm birth (19.3% vs. 10.1%) and a lower birth weight (2901 ± 625 g vs. 3063 ± 576 g) compared to the group with a sum of less than 15 cm (Table 4 and Supporting information, Table S1). Since IM type myomas were removed together with SS myomas in more than half of the cases (Table 1), the group in which myomas including IM type were removed and the group in which only SS type myomas were removed were compared. The risk of neonatal composite morbidity, including neonatal sepsis, retinopathy of prematurity, persistent ductus arteriosus, acute respiratory distress syndrome, bronchopulmonary dysplasia, and necrotizing enterocolitis, was higher in the group in which myomas including the IM type were removed than in the group in which only the SS type myomas were removed (aOR = 14.29, 95% CI [1.82–99.57], *p* = 0.012). Although not statistically significant, the group in which myomas including the IM type were removed showed a lower placental weight (683 ± 207 vs. 738 ± 213) compared to the group in which only SS type myomas were removed (Table 5). When comparing 25 cases (10.1%) of only IM type myoma and 77 cases (31.1%) of only SS type myoma, there were no statistically significant differences in obstetric outcomes (Supporting information, Table S2). Compared to cases with a longer interval between myomectomy and pregnancy (more than 24 months), those with a shorter interval (less than 24 months) had a lower placental weight (655 ± 159 g vs. 726 ± 230 g, *p* = 0.011) (Table 6). In a subgroup analysis according to the time between myomectomy and pregnancy, the frequency of placenta accreta/increta was approximately four times higher in the group with an interval of less than 12 months compared to the group with an interval of 12 months or more (Supporting information, Table S3). There was no difference in obstetric or neonatal outcomes according to the surgical method (laparoscopy vs. laparotomy) for myomectomy (Supporting information, Table S4).

**Table 1:** Clinical characteristics of the study population

	Delivery after pre-pregnancy myomectomy(*N*=248)
**Clinical characteristics in pregnancy**	
Maternal age (y)^a^	34.9 ± 3.8
Nulliparity^b^	182 (73.4)
Height (cm)^a^	161.5 ± 5.1
BMI at the first visit for the antenatal care^a^	23.0 ± 4.3
Spontaneous abortion after myomectomy^b^	32 (12.9)
Method of conception	
Spontaneous^b^	187 (75.4)
Ovarian stimulation^b^	6 (2.4)
In vitro fertilization^b^	55 (22.2)
Twin pregnancy^b^	18 (7.3)
Interval time between myomectomy and pregnancy (month)^a^	23.8 ± 23.2
<6 months^b^	46 (18.5)
≤6 and <12 months^b^	43 (17.3)
≤12 and <24 months^b^	76 (30.6)
≥24 months^b^	83 (33.5)
**Clinical characteristics at the time of myomectomy**	
Age (y)^a^	32.9 ± 4.1
BMI at the time of myomectomy^a^	22.0 ± 3.2
Spontaneous abortion before myomectomy^b^	69 (27.8)
Prior preterm birth before myomectomy^b^	4 (1.6)
Number of uterine myomas removed	
One^b^	102 (41.1)
Two^b^	42 (16.9)
Three^b^	27 (10.9)
Four or more^b^	77 (31.0)
Sum of the diameters of which the three biggest uterine myomas^a^	11.0 ± 4.3
Type of uterine myomas	
Only intramural myomas^b^	25 (10.1)
Only subserosal myomas^b^	77 (31.0)
Coexistence of intramural and subserosal myomas^b^	146 (58.9)
Laparoscopic myomectomy^b^	70 (28.5)

*BMI* Body mass index; ^a^Data given as mean ± SD, ^b^Data given as n (%)

**Table 2:** Obstetric and neonatal outcomes

	Delivery after pre-pregnancy myomectomy (*N*=248)
Pregnancies with Preeclampsia^*^	6 (0.2)
Loss of the fetus during GA 16-23 weeks^*^	2 (0.8)
GA at delivery (week)^†^	37.7 ± 2.4
Preterm birth	
23-27^+6^ weeks^*^	1 (0.4)
23-31^+6^ weeks^*^	4 (1.6)
23-33^+6^ weeks^*^	5 (2.0)
23-36^+6^ weeks^*^	30 (12.1)
Cesarean delivery^*^	233 (94.0)
Emergent delivery^*^	43 (17.3)
Uterine rupture^*^	1 (0.4)
Declined hemoglobin after delivery (g/dl)^†^	1.6 ± 1.3
Transfusion during peripartum period^*^	67 (27.0)
Placental accreta/increta^*^	13 (5.2)
Placental weight (g)†	700.2 ± 210.2
	Neonates after 23 weeks (*N*=246)
Neonatal outcomes	
Birth weight (gram)†	3025.5 ± 590.0
SGA^*^	15 (6.0)
Apgar score at 5min <7^*^	6 (2.4)
NICU admission^*^	41 (16.5)
Neonatal death	0 (0.0)
Morbidity during hospitalization	
Use of a ventilator^*^	33 (13.4)
Respiratory distress syndrome^*^	14 (5.7)
Bronchopulmonary dysplasia^*^	1 (0.4)
Composite morbidity^a^^*^	20 (8.1)

*GA* Gestational age, *SGA* Small for gestational age, *NICU* Neonatal intensive care unit; ^a^Composite morbidity during hospitalization includes neonatal sepsis, retinopathy of prematurity, patent ductus arteriosus, respiratory distress syndrome, bronchopulmonary dysplasia and necrotizing enterocolitis; ^*^Data given as n (%), ^†^Data given as mean ± SD

**Table 3:** Obstetric outcomes according to the number of uterine myomas removed

	Number of uterine myomas removed (single)(*N*=102)	Number of uterine myomas removed (≥2)(*N*=146)	*p*-value	Unadjusted odds ratio (95% CI)(Reference: single)	*p*-value	Adjusted odds ratio (95% CI)^a^(Reference: single)	*p*-value
GA at delivery (weeks)*	37.5 ± 2.6	37.8 ± 2.2	0.351				
Birthweight (g)*	3046 ± 570	3011 ± 605	0.651				
Placental weight (g)*	718 ± 233	688 ± 192	0.257				
Preterm birth^†^	14 (13.9)	16 (11.0)	0.317	0.77 (0.36-1.66)	0.506	0.66 (0.24-1.80)	0.413
Emergent delivery^†^	16 (15.8)	27 (18.5)	0.358	1.21 (0.61-2.38)	0.589	0.93 (0.42-2.07)	0.862
Placental accreta/increta^†^	4 (3.9)	9 (6.2)	0.317	1.61 (0.48-5.38)	0.439	2.15 (0.55-8.36)	0.270
Transfusion^†^	19 (18.6)	48 (32.9)	0.009	2.14 (1.17-3.92)	0.014	2.41 (1.20-4.86)	0.014
SGA^†^	4 (3.9)	11 (7.5)	0.184	2.00 (0.62-6.46)	0.248	2.73 (0.68-10.94)	0.155
NICU admission^†^	15 (14.9)	26 (17.9)	0.324	1.25 (0.63-2.51)	0.524	1.18 (0.51-2.73)	0.707
Composite morbidity ^b^^†^	9 (8.8)	11 (7.5)	0.444	0.84 (0.34-2.11)	0.714	0.62 (0.20-1.91)	0.404

*GA* Gestational age, *SGA* Small for gestational age, *NICU* neonatal intensive care unit; ^a^All outcomes were adjusted for maternal age, body mass index, method of conception, twin pregnancy, Interval time between myomectomy and pregnancy, myomectomy method, sum of the diameters of which the three biggest uterine myomas, and type of uterine myomas;^b^Composite morbidity includes neonatal sepsis, retinopathy of prematurity, patent ductus arteriosus, respiratory distress syndrome, bronchopulmonary dysplasia and necrotizing enterocolitis; *Data given as mean ± SD, ^†^Data given as n (%)

**Table 4:** Obstetric outcomes according to the sum of the diameters of which the three biggest uterine myomas

	Sum of the diameters of which the three biggest uterine myomas (<15cm)(*N*=191)	Sum of the diameters of which the three biggest uterine myomas (≥15cm)(*N*=57)	*p*-value	Unadjusted odds ratio (95% CI)(Reference: <15cm)	*p*-value	Adjusted odds ratio (95% CI) ^a^(Reference: <15cm)	*p*-value
GA at delivery (weeks)*	37.7 ± 2.5	37.6 ± 1.9	0.805				
Birthweight (g)*	3063 ± 576	2901 ± 625	0.070				
Placental weight (g)*	697 ± 204	710 ± 232	0.699				
Preterm birth^†^	19 (10.1)	11 (19.3)	0.055	2.14 (0.95-4.81)	0.066	2.41 (0.87-6.67)	0.091
Emergent delivery^†^	30 (15.8)	13 (22.8)	0.152	1.58 (0.76-3.27)	0.223	1.17 (0.74-3.75)	0.219
Placental accreta/increta^†^	11 (5.8)	2 (3.5)	0.391	0.60 (0.13-2.77)	0.508	0.45 (0.09-2.28)	0.332
Transfusion^†^	52 (27.2)	15 (26.3)	0.519	0.96 (0.49-1.87)	0.892	0.80 (0.39-1.64)	0.533
SGA^†^	11 (5.8)	4 (7.0)	0.466	1.24 (0.38-4.04)	0.727	1.23 (0.33-4.57)	0.754
NICU admission^†^	32 (16.9)	9 (15.8)	0.510	0.92 (0.41-2.06)	0.839	0.84 (0.33-2.12)	0.710
Composite morbidity ^b^^†^	14 (7.3)	6 (10.5)	0.298	1.49 (0.54-4.06)	0.439	1.61 (0.51-5.09)	0.416

*GA* Gestational age, *SGA* Small for gestational age, *NICU* neonatal intensive care unit; ^a^All outcomes were adjusted for maternal age, body mass index, method of conception, twin pregnancy, Interval time between myomectomy and pregnancy, myomectomy method, number of uterine myomas removed, type of uterine myomas;^b^Composite morbidity includes neonatal sepsis, retinopathy of prematurity, patent ductus arteriosus, respiratory distress syndrome, bronchopulmonary dysplasia and necrotizing enterocolitis; *Data given as mean ± SD, ^†^Data given as n (%)

**Table 5:** Obstetric outcomes according to the type of uterine myomas

	Including intramural type(*N*=171)	Only subserosal type(*N*=77)	*p*-value	Unadjusted odds ratio (95% CI)(Reference: Including intramural type)	*p*-value	Adjusted odds ratio (95% CI)^a^(Reference: Including intramural type)	*p*-value
GA at delivery (weeks)*	37.7 ± 2.3	37.7 ± 2.7	0.829				
Birthweight (g)*	3032 ± 563	3011 ± 648	0.794				
Placental weight (g)*	683 ± 207	738 ± 213	0.055				
Preterm birth^†^	19 (11.2)	11 (14.5)	0.297	1.35 (0.61-2.99)	0.466	0.82 (0.29-2.27)	0.695
Emergent delivery^†^	31 (18.2)	12 (15.6)	0.377	0.83 (0.40-1.72)	0.611	0.81 (0.38-1.74)	0.597
Placental accreta/increta^†^	11 (6.4)	2 (2.6)	0.173	0.39 (0.08-1.79)	0.225	0.41 (0.08-2.10)	0.283
Transfusion^†^	45 (26.3)	22 (28.6)	0.411	1.12 (0.62-2.04)	0.711	1.47 (0.76-2.84)	0.249
SGA^†^	10 (5.8)	5 (6.5)	0.523	1.12 (0.37-3.39)	0.844	0.83 (0.23-3.03)	0.778
NICU admission^†^	30 (17.6)	11 (14.5)	0.338	0.79 (0.37-1.67)	0.538	0.65 (0.28-1.54)	0.330
Composite morbidity ^b^^†^	19 (11.1)	1 (1.3)	0.005	0.11 (0.01-0.80)	0.030	0.07 (0.01-0.55)	0.012

*GA* Gestational age, *SGA* Small for gestational age, *NICU* Neonatal intensive care unit; ^a^All outcomes were adjusted for maternal age, body mass index, method of conception, twin pregnancy, Interval time between myomectomy and pregnancy, myomectomy method, sum of the diameters of which the three biggest uterine myomas, and number of uterine myomas removed;^b^Composite morbidity includes neonatal sepsis, retinopathy of prematurity, patent ductus arteriosus, respiratory distress syndrome, bronchopulmonary dysplasia and necrotizing enterocolitis; *Data given as mean ± SD, ^†^Data given as n (%)

**Table 6:** Obstetric outcomes according to the interval time between the myomectomy and the pregnancy

	Interval time between myomectomy and pregnancy (<24 months)(*N*=89)	Interval time between myomectomy and pregnancy (≥24 months)(*N*=159)	*p*-value	Unadjusted odds ratio (95% CI)(Reference: <24 months)	*p*-value	Adjusted odds ratio (95% CI)^a^(Reference: <24 months)	*p*-value
GA at delivery (weeks)*	37.8 ± 2.4	37.7 ± 2.4	0.620				
Birthweight (g)*	3027 ± 599	3025 ± 587	0.975				
Placental weight (g)*	655 ± 159	726 ± 230	0.011				
Preterm birth^†^	7 (8.0)	23 (14.6)	0.092	2.14 (0.95-4.81)	0.066	0.99 (0.36-2.76)	0.993
Emergent delivery^†^	17 (19.1)	26 (16.5)	0.359	1.58 (0.76-3.27)	0.223	1.67 (0.66-4.18)	0.277
Placental accreta/increta^†^	7 (7.9)	6 (3.8)	0.138	0.60 (0.13-2.77)	0.508	0.27 (0.06-1.35)	0.111
Transfusion^†^	24 (27.0)	43 (27.0)	0.557	0.96 (0.49-1.87)	0.892	1.13 (0.53-2.42)	0.745
SGA^†^	6 (6.7)	9 (5.7)	0.465	1.24 (0.38-4.04)	0.727	1.02 (0.21-4.92)	0.983
NICU admission^†^	18 (20.5)	23 (14.6)	0.156	0.92 (0.41-2.06)	0.839	0.50 (0.18-1.35)	0.170
Composite morbidity ^b^^†^	8 (9.0)	12 (7.5)	0.430	1.49 (0.54-4.07)	0.439	0.56 (0.14-2.20)	0.405

*GA* Gestational age, *SGA* Small for gestational age, *NICU* Neonatal intensive care unit; ^a^All outcomes were adjusted for maternal age, body mass index, method of conception, twin pregnancy, myomectomy method, sum of the diameters of which the three biggest uterine myomas, number of uterine myomas removed, and type of uterine myomas;^b^Composite morbidity includes neonatal sepsis, retinopathy of prematurity, patent ductus arteriosus, respiratory distress syndrome, bronchopulmonary dysplasia and necrotizing enterocolitis; *Data given as mean ± SD, ^†^Data given as n (%)

There were 43 cases of emergency delivery, and the gestational age at the emergency delivery was 36.2 (26.9–40.3) weeks. The indications for emergency delivery were uterine rupture (1 case, 2.3%), preterm premature rupture of membranes (11 cases, 25.6%), and preterm labor (9 cases, 20.9%) (Supporting information, Table S5). When comparing the clinical characteristics at the time of myomectomy between elective and emergent delivery cases (except for medically indicated delivery), the interval time between myomectomy and pregnancy was significantly shorter in the case of emergent delivery compared to elective delivery (18.8 ± 14.6 months vs. 25.0 ± 24.5 months, *p* = 0.035) (Supporting information, Table S6).

## Discussion

Recently, as more women are delaying pregnancy and childbirth, the incidence of pregnancies complicated by uterine myomas or previous myomectomy has increased. The presence of a uterine myoma may have a detrimental effect on implantation by obstructing reproductive cell migration, altering uterine contractions, and adversely affecting the endometrium [5]. Most pregnant women with uterine myomas do not have complications associated with the uterine myomas. Abdominal or pelvic pain may present in the late first or early second trimester, and there may be a slightly increased risk of obstetric complications, such as miscarriage, preterm birth, fetal malpresentation, fetal growth restriction, and placental abruption, especially in women with multiple uterine myomas, and size greater than 5 cm [6, 7].

Increased use of ultrasound imaging has led to more diagnoses of asymptomatic uterine myomas, and some early studies of women with unknown causes of infertility reported improved pregnancy rates in women with uterine myomas after myomectomy; however, these studies had certain limitations including the lack of randomization methods, and not considering other factors which may influence pregnancy outcomes [8]. In our study, the main reasons for deciding on myomectomy were a recommendation that removal of uterine myomas will help for pregnancy, which was the most common with 101 (40.7%), followed by a rapid increase in the size of fibroids with 74 (29.8%), anemia due to menorrhagia was 59 (23.8%) and uncomfortable symptom due to mass effect was 14 (5.6%). These results are similar to those reported in previous studies [9]. Although there are reports that myomectomy is a safe treatment option for women preparing for pregnancy [10], it is unclear whether this procedure offers a therapeutic benefit for women of childbearing age. Several factors should be considered when deciding whether to perform myomectomy, including the size and number of myomas, and their location in the uterus. Although several studies have shown that patient age, myoma size, myoma location, and the interval between myomectomy and pregnancy may influence pregnancy and live birth rates after myomectomy [11–13], to our knowledge, this is the first study to investigate the association between clinical features at the time of myomectomy before pregnancy and various adverse obstetric and perinatal outcomes.

Normal uterine myometrium is damaged during myomectomy. Removal of IM type myomas results in scarring of the uterine muscle; when the muscular tissue regenerates, the healing process consists of the regeneration of muscle fibers and formation of a connective tissue scar. This scar may interfere with decidual reactions, spiral artery remodeling, and successful placentation. Uterine spiral arteries play a vital role in supplying nutrients to the placenta and fetus, and for this purpose, they are remodeled into highly dilated vessels by invasion [14]. If this process fails, vascular malperfusion and its relationship with oxygen, trophoblast invasion from the outside or inside of the vessels (intravasation versus extravasation), the impact of hemodynamics on endovascular migration, the replacement of arterial components by trophoblasts, maternal tissue repair mechanisms, and the role of uterine natural killer cells can arise, which can lead to various obstetrical and perinatal complications [14]. Eventually, placental malperfusion may occur, and this pathological condition may manifest as a small placenta. Placentation near the post-procedure myomectomy site impairs intervillous blood flow [15], resulting in uteroplacental insufficiency and fetal growth restriction. Previous studies have shown that placental volume at delivery is associated with adverse pregnancy outcomes [16, 17].

In our study, the risk of neonatal composite morbidity, including neonatal sepsis, retinopathy of prematurity, persistent ductus arteriosus, acute respiratory distress syndrome, bronchopulmonary dysplasia, and necrotizing enterocolitis, was higher in the group in which myomas including the IM type were removed than in the group in which only the SS type myomas were removed. Although not statistically significant, the group in which myomas, including the IM type, were removed, showed a lower placental weight compared to the group in which only SS type myomas were removed. This suggests that damage to the vascular tissue of the myometrium or replacement of fibrotic tissue in the normal myometrium can adversely affect the growth of the placenta, the development of the fetus, and the maturation of fetal organs including the cardiovascular system, lungs, bowels, eyes, etc. Although not statistically significant, the group in which the sum of the diameters of three largest myomas was greater than 15 cm showed a lower birth weight compared to the group with a total diameter less than 15 cm. The results of our study suggest that the severity of the myometrial scar after myomectomy may affect future pregnancy outcomes.

Disorders in placental formation and growth are also associated with preterm births. Oxidative stress due to insufficient uteroplacental perfusion can lead to preterm labor and preterm birth [18, 19]. Previous studies have shown an association between a history of myomectomy and preterm birth [20–24]. In this study, the preterm birth rate in women with a history of myomectomy was 12.1% (30 women), which appears to be slightly higher than the incidence in the normal standard population. Although not statistically significant, a higher frequency of preterm birth (19.3% vs. 10.1%) was observed in the group with a sum of diameters of the three largest myomas greater than 15 cm compared to less than 15 cm.

Previous reports have shown that most surgeons recommend contraception for 6–24 months after myomectomy [11]. It takes time for the injured myometrium to recover after myomectomy; if pregnancy occurs before the damaged myometrium fully heals, the placenta may invade the myometrium at the site of the resection, which can cause abnormal placentation. Placenta accreta or increta are serious complications which can result in severe maternal hemorrhage [25, 26]. In our study, a shorter interval between myomectomy and pregnancy (less than 12 months) was associated with a higher frequency of placenta accreta or increta compared to an interval of more than 12 months. Insufficient recovery of the myometrium can also affect placental growth [27]. Accordingly, in our study, the placental weight was lower when the interval between myomectomy and subsequent pregnancy was less than 2 years. This study also showed that the shorter the interval time between myomectomy and subsequent pregnancy, the higher the risk of emergency delivery. The reasons for emergency delivery were preterm labor or preterm premature rupture of membranes, and uterine rupture, which, as described above, are likely to be related to insufficient recovery of the myomectomy site.

The strengths of this study are mostly related to the homogeneity of data, as they were collected in a single center, and two experienced expert operators performed the myomectomy procedure. Both surgeons used the same suture materials and methods for myometrial reapproximation. However, this study was limited by its retrospective design, which precluded us from following up all women who underwent myomectomy. Although this study included a relatively large number of patients compared to previous studies, in the future, larger prospective controlled studies are needed to validate our results and obtain sufficient data to reliably inform patients about the risks of myomectomy before pregnancy and counsel pregnant women about the increased risk of perinatal complications caused by myomectomy.

## Conclusions

In conclusion, if the removed myomas are multiple, IM, large, or the interval between myomectomy and pregnancy is short, the risk of obstetric and neonatal complications may increase. These findings are of clinical significance, and they may help obstetricians to effectively counsel pregnant women with a history of myomectomy regarding the risk of subsequent pregnancies.

## Supplementary Information

Additional file 1: **Table S1.** Obstetric outcomes according to the sum of the diameters of which the three biggest uterine myomas. **Table S2. **Obstetric outcomes between the cases with only intramural myomas and only subserosal myomas. **Table S3. **Obstetric outcomes according to the interval time between the myomectomy and the pregnancy. **Table S4. **Obstetric outcomes according to the myomectomy method. **Table S5. **Cases of emergent delivery. **Table S6. **Comparison of clinical characteristics between elective and emergent delivery cases (except for medically indicated delivery).

## Data Availability

The datasets generated and/or analyzed during the current study are not publicly available due to concerns about the risk of data being used outside for other purposes but are available from the corresponding author on reasonable request.

## Abbreviations

BMI: Body mass index
IM: Intramural
SS: Subserosal
