# Supplementary material for: Clinical features at the time of non-hysteroscopic myomectomy before pregnancy, which affect adverse pregnancy outcomes: a retrospective cohort study
Source: BMC Pregnancy Childbirth. 2022 Dec 3;22:896. doi: 10.1186/s12884-022-05240-7 (PMC9719619; doi:10.1186/s12884-022-05240-7)
Supplement: Supplementary file 1 — Table S1. Obstetric outcomes according to the sum of the diameters of which the three biggest uterine myomas. Table S2. Obstetric outcomes between the cases with only intramural myomas and only subserosal myomas. Table S3. Obstetric outcomes according to the interval time between the myomectomy and the pregnancy. Table S4. Obstetric outcomes according to the myomectomy method. Table S5. Cases of emergent delivery. Table S6. Comparison of clinical characteristics between elective and emergent delivery cases (except for medically indicated delivery). [file 12884_2022_5240_MOESM1_ESM.docx]

**Table S1.** Obstetric outcomes according to the sum of the diameters of which the three biggest uterine myomas.

|  | Sum of the diameters of which the three biggest uterine myomas  (<5cm)  (N=10) | Sum of the diameters of which the three biggest uterine myomas  (≤5 and <10cm)  (N=91) | Sum of the diameters of which the three biggest uterine myomas  (≤10 and <15cm)  (N=90) | Sum of the diameters of which the three biggest uterine myomas  (≤15cm)  (N=57) | p-value |
| --- | --- | --- | --- | --- | --- |
| GA at delivery (weeks)* | 37.5 ± 2.9 | 37.3 ± 3.4 | 38.2 ± 1.1 | 37.6 ± 1.9 | 0.111 |
| Birthweight (g)* | 2896 ± 528 | 3018 ± 707 | 3126 ± 406 | 2901 ± 625 | 0.131 |
| Placental weight (g)* | 654 ± 198 | 682 ± 198 | 717 ± 211 | 710 ± 232 | 0.601 |
| Preterm birth* | 1 (10.0) | 11 (12.4) | 7 (7.8) | 11 (19.3) | 0.224 |
| Emergent delivery^†^ | 1 (10.0) | 14 (15.6) | 15 (16.7) | 13 (22.8) | 0.618 |
| Placental accreta/increta^†^ | 0 (0.0) | 7 (7.7) | 4 (4.4) | 2 (3.5) | 0.549 |
| Transfusion^†^ | 2 (20.0) | 24 (26.4) | 26 (28.9) | 15 (26.3) | 0.931 |
| SGA^†^ | 1 (10.0) | 8 (8.8) | 2 (2.2) | 4 (7.0) | 0.273 |
| NICU admission^†^ | 3 (30.0) | 11 (12.4) | 18 (20.0) | 9 (15.8) | 0.359 |
| Composite morbidity ^b†^ | 1 (10.0) | 3 (3.3) | 10 (11.1) | 6 (10.5) | 0.218 |

GA, gestational age; SGA, small for gestational age; NICU, neonatal intensive care unit; ; ^a^ All outcomes were adjusted for maternal age, body mass index, method of conception, twin pregnancy, Interval time between myomectomy and pregnancy, myomectomy method, number of uterine myomas removed, type of uterine myomas; ^b^ Composite morbidity includes neonatal sepsis, retinopathy of prematurity, patent ductus arteriosus, respiratory distress syndrome, bronchopulmonary dysplasia and necrotizing enterocolitis; *Data given as mean ± SD, ^†^ Data given as n (%)

**Table S2.** Obstetric outcomes between the cases with only intramural myomas and only subserosal myomas.

|  | Only intramural type  (N=25) | Only subserosal type  (N=77) | p-value |
| --- | --- | --- | --- |
| GA at delivery (weeks)* | 38.3 (34.3-39.4) | 38.3 (23.1-41.3) | 0.221 |
| Birthweight (g)* | 3130 (1310-3990) | 3020 (255-4320) | 0.354 |
| Placental weight (g)* | 675 (303-1249) | 670 (175-1390) | 0.778 |
| Preterm birth^†^ | 4 (16.0) | 11 (14.5) | 0.539 |
| Emergent delivery^†^ | 5 (20.0) | 12 (15.6) | 0.405 |
| Placental accreta/increta^†^ | 1 (4.0) | 2 (2.6) | 0.574 |
| Transfusion^†^ | 6 (24.0) | 22 (28.6) | 0.434 |
| SGA^†^ | 1 (4.0) | 5 (6.5) | 0.543 |
| NICU admission^†^ | 4 (16.0) | 11 (14.5) | 0.539 |
| Composite morbidity ^b†^ | 2 (8.0) | 1 (1.3) | 0.148 |

GA, gestational age; SGA, small for gestational age; NICU, neonatal intensive care unit; *Data given as median (range), ^†^ Data given as n (%)

**Table S3.** Obstetric outcomes according to the interval time between the myomectomy and the pregnancy.

|  | <6 months  (N=46) | ≤6 and <12 months  (N=43) | ≤12 and <24 months  (N=76) | ≥24 months  (N=83) | p-value |
| --- | --- | --- | --- | --- | --- |
| GA at delivery (weeks)* | 38.1 ± 1.2 | 37.5 ± 3.2 | 37.5 ± 2.9 | 37.8 ± 1.8 | 0.600 |
| Birthweight (g)* | 3034 ±464 | 3020 ± 721 | 2962 ± 622 | 3082 ± 550 | 0.652 |
| Placental weight (g)* | 663 ± 150 | 646 ± 170 | 750 ± 232 | 703 ± 209 | 0.034 |
| Preterm birth^†^ | 4 (8.7) | 3 (7.1) | 11 (14.7) | 12 (14.5) | 0.503 |
| Emergent delivery^†^ | 8 (18.6) | 9 (20.9) | 16 (21.1) | 10 (12.2) | 0.453 |
| Placental accreta/increta^†^ | 1 (2.2) | 6 (14.0) | 3 (3.9) | 3 (3.6) | 0.043 |
| Transfusion^†^ | 13 (28.3) | 11 (25.6) | 20 (26.3) | 23 (27.7) | 0.989 |
| SGA^†^ | 2 (4.3) | 4 (9.3) | 3 (3.9) | 6 (7.2) | 0.609 |
| NICU admission^†^ | 10 (21.7) | 8 (19.0) | 11 (14.7) | 12 (14.5) | 0.675 |
| Composite morbidity ^a†^ | 4 (8.7) | 4 (9.3) | 4 (5.3) | 8 (9.6) | 0.754 |

GA, gestational age; SGA, small for gestational age; NICU, neonatal intensive care unit; ^a^ Composite morbidity includes neonatal sepsis, retinopathy of prematurity, patent ductus arteriosus, respiratory distress syndrome, bronchopulmonary dysplasia and necrotizing enterocolitis; *Data given as mean ± SD, ^†^ Data given as n (%)

**Table S4.** Obstetric outcomes according to the myomectomy method.

|  | Laparoscopy  (N=70) | Laparotomy  (N=178) | p-value | Unadjusted odds ratio (95% CI)  (Reference: Laparoscopy) | p-value | Adjusted odds ratio (95% CI) ^a^  (Reference: Laparoscopy) | p-value |
| --- | --- | --- | --- | --- | --- | --- | --- |
| GA at delivery (weeks)* | 38.0 ± 1.5 | 37.6 ± 2.7 | 0.239 |  |  |  |  |
| Birthweight (g)* | 3126 ± 511 | 2986 ± 615 | 0.094 |  |  |  |  |
| Placental weight (g)* | 702 ± 155 | 700 ± 229 | 0.933 |  |  |  |  |
| Preterm birth^†^ | 5 (7.1) | 25 (14.2) | 0.091 | 2.15 (0.79-5.87) | 0.134 | 2.62 (0.74-9.26) | 0.136 |
| Emergent delivery^†^ | 11 (15.7) | 32 (18.1) | 0.406 | 1.18 (0.56-2.50) | 0.659 | 1.05 (0.48-2.33) | 0.900 |
| Placental accreta/increta^†^ | 3 (4.3) | 10 (5.6) | 0.475 | 1.33 (0.36-4.98) | 0.673 | 1.49 (0.34-6.41) | 0.596 |
| Transfusion^†^ | 18 (25.7) | 49 (27.5) | 0.452 | 1.10 (0.59-2.06) | 0.772 | 1.24 (0.63-2.476) | 0.535 |
| SGA^†^ | 3 (4.3) | 12 (6.7) | 0.345 | 1.61 (0.44-5.90) | 0.469 | 2.49 (0.47-13.09) | 0.282 |
| NICU admission^†^ | 14 (20.0) | 27 (15.3) | 0.241 | 0.73 (0.36-1.48) | 0.378 | 0.65 (0.29-1.47) | 0.303 |
| Composite morbidity ^b†^ | 6 (8.6) | 14 (7.9) | 0.517 | 0.91 (0.34-2.47) | 0.854 | 0.51 (0.17-1.56) | 0.236 |

GA, gestational age; SGA, small for gestational age; NICU, neonatal intensive care unit; ; ^a^ All outcomes were adjusted for maternal age, body mass index, method of conception, twin pregnancy, Interval time between myomectomy and pregnancy, sum of the diameters of which the three biggest uterine myomas, number of uterine myomas removed, and type of uterine myomas; ^b^ Composite morbidity includes neonatal sepsis, retinopathy of prematurity, patent ductus arteriosus, respiratory distress syndrome, bronchopulmonary dysplasia and necrotizing enterocolitis; *Data given as mean ± SD, ^†^ Data given as n (%)

**Table S5.** Cases of emergent delivery.

|  | Emergent delivery  (N=43) |
| --- | --- |
| GA at emergent delivery (week)* | 36.2 (26.9-40.3) |
| Reason of emergent delivery |  |
| Uterine rupture^†^ | 1 (2.3) |
| PPROM^†^ | 11 (25.6) |
| Preterm labor^†^ | 9 (20.9) |
| PROM (full-term)^†^ | 9 (20.9) |
| Labor (full-term)^†^ | 9 (20.9) |
| Preeclampsia^†^ | 4 (9.3) |

GA, gestational age; PPROM, preterm premature rupture of membrane; PROM, premature rupture of membrane; *Data given as mean ± SD, ^†^ Data given as mean (range)

**Table S6.** Comparison of clinical characteristics between elective and emergent delivery cases (except for medically indicated delivery).

|  | Emergent delivery  (N=39) | Elective delivery  (N=204) | p-value |
| --- | --- | --- | --- |
| Maternal age (y)* | 34.7 ± 3.6 | 34.9 ± 3.9 | 0.721 |
| BMI* | 22.9 ± 3.6 | 23.0 ± 4.5 | 0.871 |
| Method of conception |  |  | 0.612 |
| Spontaneous^†^ | 30 (76.9) | 183 (75.4) |  |
| Ovarian stimulation^†^ | 0 (0.0) | 5 (2.5) |  |
| In vitro fertilization^†^ | 9 (23.1) | 45 (22.2) |  |
| Twin pregnancy^†^ | 3 (7.0) | 15 (7.4) | 0.615 |
| Number of uterine myomas removed |  |  | 0.559 |
| One^†^ | 16 (41.0) | 85 (41.7) |  |
| Two^†^ | 8 (20.5) | 34 (16.7) |  |
| Three^†^ | 6 (15.4) | 20 (9.8) |  |
| Four or more^†^ | 9 (34.1) | 65 (31.9) |  |
| Sum of the diameters of which the three biggest uterine myomas* | 11.4 ± 4.3 | 10.9 ± 4.3 | 0.467 |
| Type of uterine myomas - only subserosal myomas^†^ | 12 (31.8) | 65 (31.9) | 0.527 |
| Laparoscopic myomectomy^†^ | 11 (28.2) | 59 (28.9) | 0.548 |
| Interval time between myomectomy and pregnancy (month)* | 18.8 ± 14.6 | 25.0 ± 24.5 | 0.035 |
| <6 months^†^ | 6 (15.4) | 38 (18.6) |  |
| ≤6 and <12 months^†^ | 8 (20.5) | 34 (16.7) |  |
| ≤12 and <24 months^†^ | 15 (38.5) | 60 (29.4) |  |
| ≥24 months^†^ | 10 (25.6) | 72 (35.3) |  |

BMI, body mass index; GA, gestational age; *Data given as mean ± SD, ^†^Data given as n (%)
